# Degenerative central lumbar spinal stenosis: is endoscopic decompression through bilateral transforaminal approach sufficient?

**DOI:** 10.1186/s12891-020-03722-3

**Published:** 2020-10-31

**Authors:** Bin Zhang, Qingquan Kong, Yuqing Yan, Pin Feng

**Affiliations:** 1 Department of Orthopedics, West China Hospital, Sichuan University, Chengdu, Sichuan China; 2 Department of Orthopedics, Hospital of Chengdu Office of People’s Government of Tibetan Autonomous Region, Chengdu, Sichuan China

**Keywords:** Central lumbar spinal stenosis, Percutaneous endoscopic lumbar decompression, Foraminoplasty, Minimally invasive treatment

## Abstract

**Background:**

At present, few reports of percutaneous endoscopic transforaminal decompression surgery have been reported to solve central lumbar spinal stenosis (CLSS). Is endoscopic decompression through bilateral transforaminal approach decompression sufficient for degenerative CLSS?

**Methods:**

This retrospective study included 47 cases of CLSS patients who underwent percutaneous endoscopic decompression through bilateral transforaminal approach. Clinical outcomes such as ODI, back and leg VAS, the Macnab criteria were evaluated. Surgical results including operative time, postoperative hospital stay, recurrence, and surgical complications were also studied. Radiologically, lumbar stability was assessed and lumbar dural sac dimension was compared preoperatively and postoperatively.

**Results:**

All 47 patients were followed up. The average follow-up period was 24.5 months. The average operation time was 116 min. The mean VAS of leg and back pain, and the mean ODI improved from 7.81, 2.53, and 77.03% at baseline to a final 1.94 (*P* = 0.00), 2.47 (*P* = 0.71), and 19.40% (*P* = 0.00), respectively. According to the Macnab criteria, 97.9% of patients achieved excellent and good results. There were 2 cases of dural tear and 3 cases of transient postoperative dysthesia. The cross-sectional area of the dural sac was significant enlargement at the last fellow up (74.28 ± 13.08 mm^2^ vs.104.91 ± 12.40 mm^2^, *P* = 0.00).

**Conclusions:**

Except for the main pathogenic factors on the dorsal side of the dural sac, percutaneous endoscopic decompression through a bilateral transforaminal approach is sufficient for CLSS. It is a feasible, safe, and clinically effective minimally invasive procedure.

## Background

Central lumbar spinal stenosis (CLSS) is a progressive degenerative disease, most commonly in patients over 60 years. CLSS is usually caused by arthritis of the facet joint, ligament hypertrophy and calcification, and disc herniation, which can significantly affect patients’ quality of life and daily activities and lead to progressive disability [1, 2]. Neurogenic claudication is the main symptom, which can be exacerbated by standing walking and relieved by lying flat and flexion of the lower back. Patients may also have tingling, numbness and weakness in the lower extremities [2–4].

Conservative management is the preferred treatment for most CLSS patients, including physical therapy, exercise therapy, analgesics, and epidural block [5, 6]. If conservative treatment fails, decompressive surgery should be considered [7, 8]. Surgical treatment of degenerative CLSS usually involves decompression nerve structure with or without fusion [9]. Conventional laminectomy provides adequate decompression by removing posterior structures including lamina, spinous processes, interspinous ligaments, ligamentum flavum and part of the facet joints [10]. However, this method has the disadvantages of chronic low back pain and iatrogenic instability and sometimes requires a second operation [11, 12]. In recent years, minimally invasive surgery (MIS) represented by micro-endoscopic decompression (MED) has achieved good clinical results in the treatment of CLSS, and some studies have revealed that these MIS techniques have obvious advantages over traditional laminectomy [11, 13].

With the rapid development of MIS, percutaneous endoscopic techniques have achieved satisfactory clinical results in disc herniation of cervical and lumbar spine. Moreover, some studies have applied percutaneous endoscopic decompression techniques to CLSS. Bilateral decompression through the unilateral interlaminar approach is considered to be a simple, safe and effective surgical procedure for CLSS [14–16]. Percutaneous endoscopic transforaminal decompression (PETD) has proven to be very useful for intervertebral foramen and lateral recess stenosis [17, 18]. But some researchers have a negative attitude toward transforaminal decompression for CLSS [19, 20]. At present, few PETD surgery has been reported to therapy CLSS in the literature. Is endoscopic decompression through lateral transforaminal approach sufficient for CLSS?

In this study, 47 cases of CLSS were collected and analyzed. Bilateral lumbar spinal decompression was performed though a percutaneous endoscopic bilateral transforaminal approach. Our goal was to evaluate the outcome and efficacy of percutaneous endoscopic transforaminal bilateral decompression for the treatment of CLSS.

## Methods

### Patients

A retrospective review was performed from February 2016 to April 2018, 47 patients of CLSS underwent percutaneous endoscopic bilateral decompression through bilateral transforaminal approach in our hospital. The inclusion criteria were: 1) Imaging examinations showed CLSS, and the dural sac area (DSA) on the cross-sectional image of magnetic resonance imaging (MRI) was less than 100 mm^2^ [21]; 2) Neurogenic intermittent claudication and bilateral lower extremity radiculopathy (buttock and lower extremity pain); 3) Back pain on the visual analogue scale (VAS) was less than 3; 4) Conservative treatment failed for at least 3 months. Exclusion criteria were: 1) Developmental lumbar spinal stenosis; 2) Isolated lateral (recess or foraminal) lumbar spinal stenosis; 3) Lumbar segmental instability, and degenerative spondylolisthesis exceeded than Meyerding I; 4) Pathological conditions (infection/tumors/fractures); 5) Patients with severe illness cannot tolerate local anesthesia; 6) Incomplete information or lost to the follow-up during follow-up period.

### Surgical tools

**Fig. 1:**
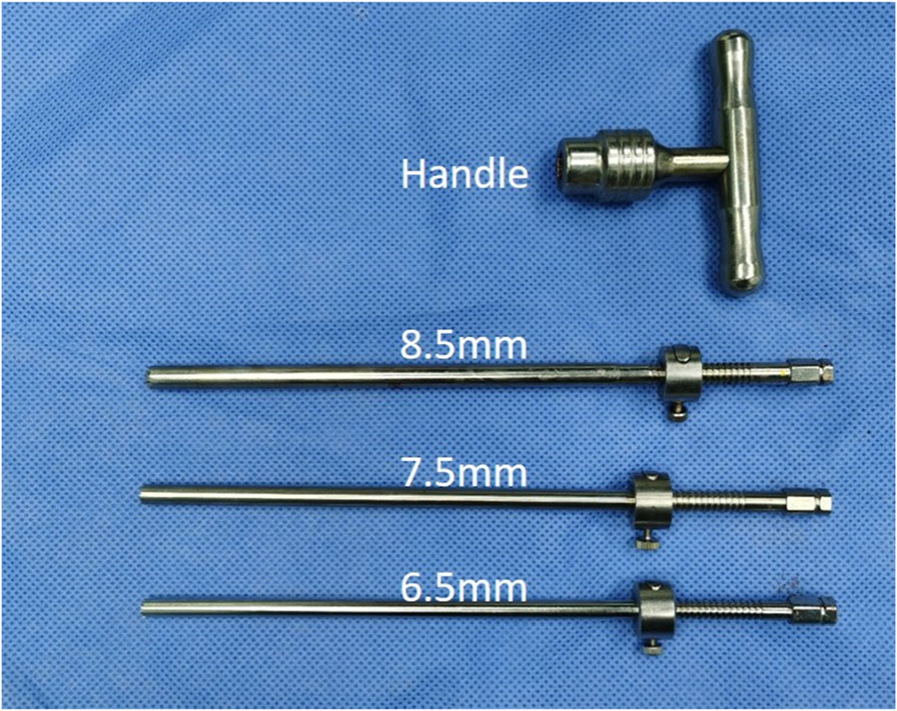
Specially designed deep-restricted trephine

An endoscopic system (Spinendos, Germany) was used for the endoscopic operation. The bone structure was removed using a high-speed drilling system (Spinendos, Germany). Bipolar radiofrequency coagulator (Elliquence, USA) was used for hemostasis and soft tissue ablation. The specially designed deep-restricted trephine (Fig. 1) had a stepped spacing of 2 mm to control the depth of entry, which allowed for more safe and effective removal of bone. According to different parts of foraminoplasty, deep-restricted trephines with diameters of 8.5 mm, 7.5 mm and 6.5 mm were available. The usage of deep-restricted trephine reduced radiation exposure and allowed for safer and more effective removal of bone structures, which could significantly reduce operative time.

### Surgical procedures

**Fig. 2:**
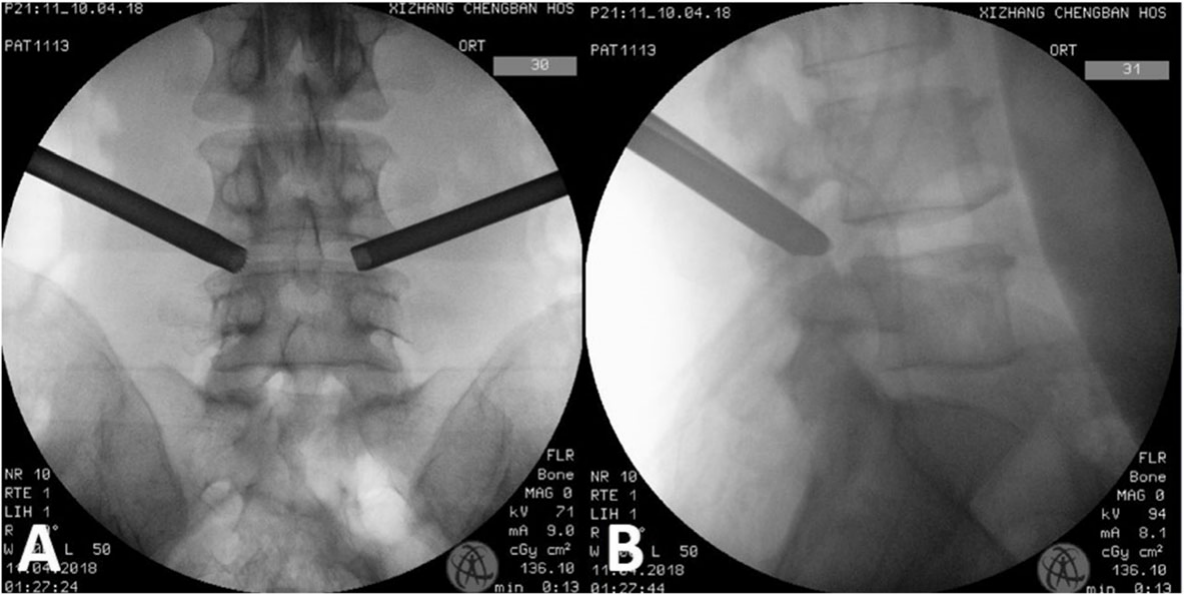
The working channel position of bilateral foraminoplasty. **a** X-ray anteroposterior view, **b** X-ray lateral view

All procedures were basically classic Thessys technology. The C-arm fluoroscopy was used to perform surgery in a prone position on a radio-permeable table under local anesthesia. Previous techniques have described techniques for skin entry points and surgical trajectories [22, 23]. An 18G needle was inserted into the lateral aspect of the facet joint, and 20 ml of 0.5% lidocaine was infiltrated into the area around the facet joint and the intervertebral foramen. A 2 mm diameter guide rod was inserted into the ventral side of the superior articular process in the safe triangle. Lateral and anteroposterior fluoroscopy confirmed the position of the guide rod. Sequential protective cannulas were introduced over the guide rod, and a 9.5 mm diameter protective cannula was inserted. The C-arm confirmed that the protective cannula was in the proper position. Foraminoplasty was performed using an 8.5 mm diameter deep-restricted trephine. Rotated carefully under fluoroscopic guidance to make the deep-restricted trephine enter. After the trephine reached a depth of 10 mm, it was gradually screwed in at intervals of 2 mm, and partial facet joint (the ventral portion of the superior articular process and the lower articular process) could be removed with the trephine once they were cut off. The contralateral foraminoplasty is performed simultaneously by two surgeons separately according to the method mentioned above, which can significantly reduce the operative time (Fig. 2).

The soft tissue blocking the visual field was cleaned under endoscope, and the bleeding was controlled by a bipolar radiofrequency electrocoagulator to expose local anatomical structures such as the facet joints, ligamentum flavum, dural sac and the nerve roots. The second foraminoplasty was performed with the deep-restricted trephine combined with a high-speed drill to expand the intervertebral foramen fully. A 7.5 mm trephine was chosen to enlarge the lateral recess or the dorsal area of the intervertebral foramen. The depth of the trephine was determined according to the thickness of the bone that needs to be removed. After the trephine removed most of the bone, a high-speed drill was used to remove the remaining bone, usually the inner side of the articular process adjacent to the spinal canal. The size of the intervertebral foramen after the second foraminoplasty was checked under the endoscope. If necessary, the third foraminoplasty was performed with a 7.5 mm or 6.5 mm depth-limiting trephine. Since the intervertebral foramen was fully enlarged, additional procedures were easy to complete, such as adjusting the cannula to make it more horizontal, tilting down or upward, so that the herniated disc, hypertrophic ligament and osteophytes could be directly observed and removed. We performed the decompression of the lateral recess, the dorsal side of the dural sac, and the ventral side of the dural sac in sequence. After the decompression was completed on one side, the other side was decompressed according to the above procedure.

**Fig. 3:**
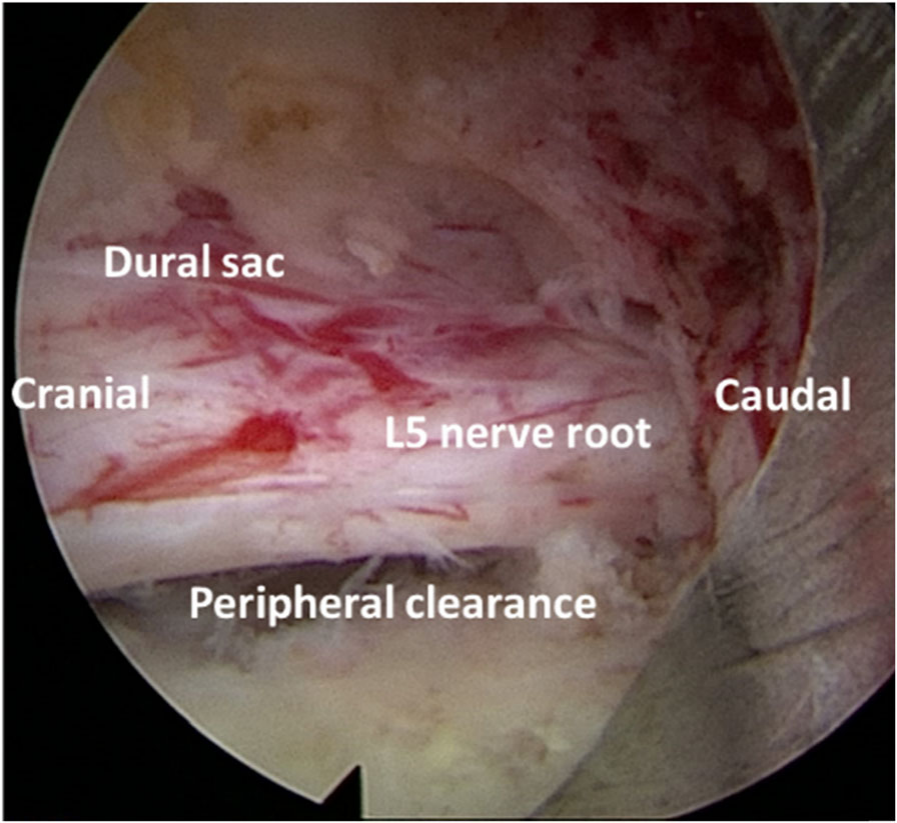
The nerve root returned to normal morphology with clear and adequate peripheral clearance

Signs of decompression completion: the dural sac is well filled, and normal pulsation is restored; the nerve roots in the lateral recess area return to normal morphology, the distal decompression reaches the upper bony lateral recess (zone 2) [23]; The dural sac and nerve roots have clear and adequate peripheral clearance (Fig. 3).

All patients underwent postoperative X-ray and MRI/CT examination 1 day after surgery. Wear soft waist protection for 6 weeks, avoid excessive physical activity and intense physical exercise within 3 months after surgery.

### Assessment of outcome

Clinical outcomes, such as the Oswestry Disability Index (ODI) [24], VAS for leg and back pain, and the Macnab criteria were evaluated. Surgical results, including operative time, postoperative hospital stay, recurrence, and surgical complications were also studied. Radiologically, lumbar stability was assessed by functional X-rays, lumbar dural sac dimension was compared preoperatively and postoperatively by cross-sectional MRI at the mid-disc level.

Statistical analysis of the comparison between preoperative and postoperative clinical outcomes was performed using repeated-measures analysis of variance and rank-sum test. A positive significance level is set at a probability value of less than 0.05.

## Results

All 47 patients were followed up for 19 to 45 months (average 24.5 months). Of 47 patients, there were 29 male and 18 female patients. Patients were between 56 and 89 years old, with an average age of 72.6 ± 9.5 years. Their mean symptom duration was 48 weeks (range 16–148). Thirty-eight patients were complicated with hypertension (24 cases), coronary heart disease (9 cases), diabetes (12 cases), chronic obstructive pulmonary disease (11 cases), arrhythmias (3 cases), heart failure (4 cases), and other medical disorders (8 cases). The affected lumbar segments were 9 in L3/4, 32 in L4/5, and 6 in L5/S1.

**Fig. 4:**
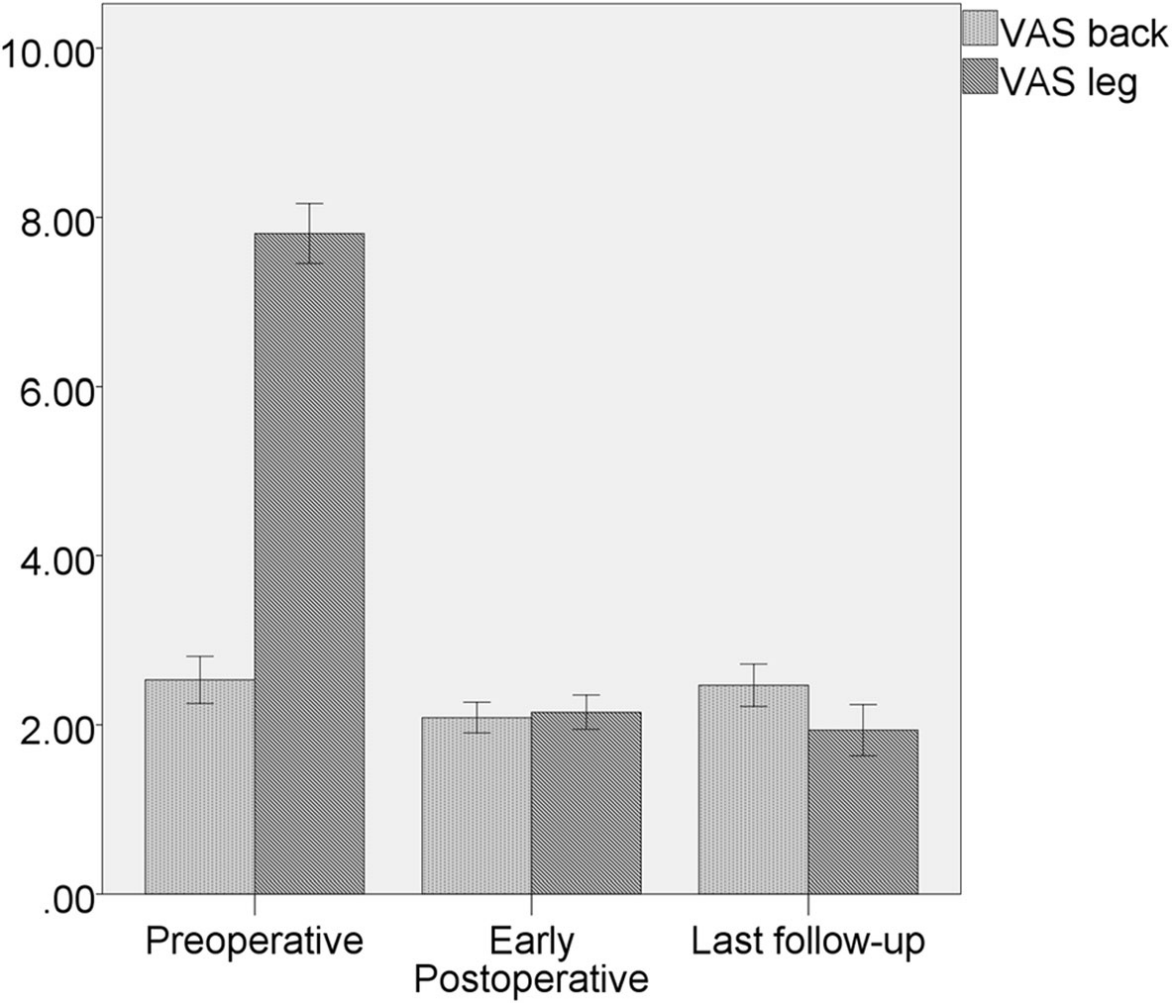
Changes in visual analogue scale (VAS)

The VAS score for preoperative back pain was 2.53 ± 0.95, which improved to 2.08 ± 0.62 at 4 weeks after surgery, and 2.47 ± 0.86 at last follow-up (*P*>0.05 respectively). The VAS score for preoperative leg pain was 7.81 ± 1.21, which improved to 2.14 ± 0.69 at 4 weeks postoperatively, and 1.94 ± 1.03 at last follow-up (*P*<0.00 respectively) (Fig. 4). The mean operative time was 116.4 ± 36.8 min, and the average hospital stay after surgery was 2.6 ± 0.9 days (range 2–4) days. The preoperative ODI was 77.03 ± 7.78. The score improved to 19.40 ± 6.40 at last follow-up (*P*<0.00). Forty six patients (97.9%) had good-to-excellent Macnab grade (Table 1). Three patient developed postoperative dysesthesia and were treated with the anti-neuropathic agent, and symptoms had improved after 1–2 months. Two cases of dural sac tear occurred on the lateral side. Due to severe stenosis, the ligamentum flavum and the dural sac adhered closely. When the rongeur was used to remove the ligamentum flavum, tears of 1 mm and 2 mm were caused respectively. Both dural tears were small cracks without special treatment. All patients had no cerebrospinal fluid leakage, hematoma, infection, recurrence, or required for revision surgery during the follow-up.

**Table 1:** Details of Outcome by Macnab Criteria

Macnab Grade	n	(%)
Poor	0	0
Fair	1	2.1
Good	17	36.2
Excellent	29	61.7

**Fig. 5:**
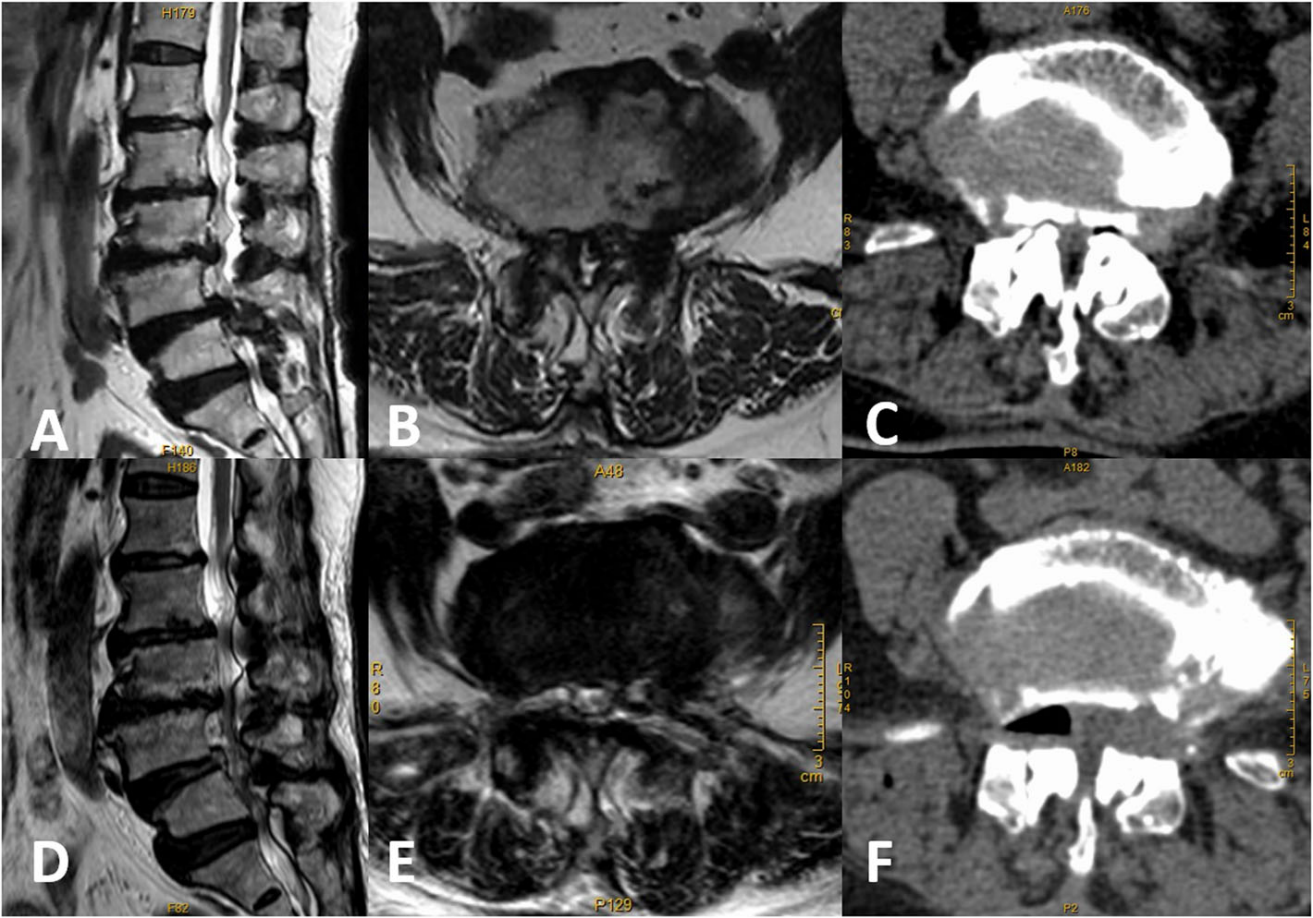
**a-b** Central spinal stenosis of L4–5 was shown in the preoperative MRI, the cross-sectional area of the dural sac was 27.16mm^2^; **c** Preoperative CT showing bilateral bony lateral recess stenosis; **d-e** Postoperative MRI showing that the central spinal canal was decompressed, the cross-sectional area of the dural sac was 98.86mm^2^; **f** Postoperative CT showing that the distal decompression reached the upper bony lateral recess (zone 2) and both the facet joints were well preserved

The postoperative MRI and CT scans showed adequate decompression in this study. The cross-sectional area of the dural sac was significantly enlarged at the last follow-up. (74.28 ± 13.08 mm^2^ vs.104.91 ± 12.40 mm^2^, *P*<0.00) (Fig. 5).

## Discussion

**Fig. 6:**
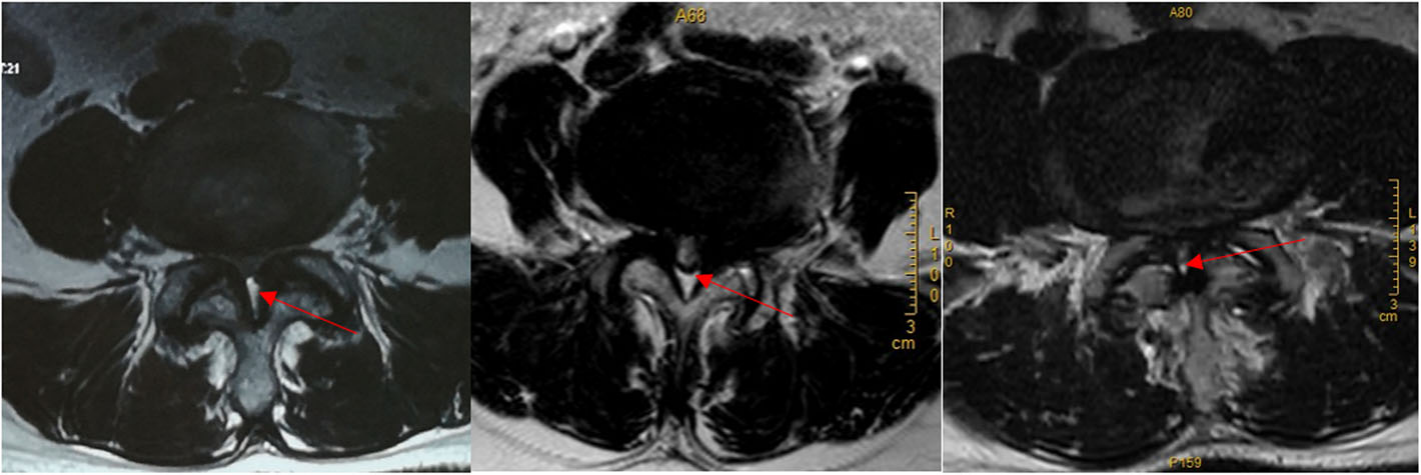
Even for severe lumbar spinal stenosis, adipose tissue can still be seen on the dorsal side of the dural sac in axial MRI images (Red arrow: adipose tissue). The pathogenic factors of stenosis mainly come from bilateral and ventral compression

Different from congenital pathological factors of developmental lumbar spinal stenosis [3, 25, 26]. Degenerative CLSS is caused by hypertrophic facet joints, hypertrophy and ossification of the ligamentum flavum, and disc herniation [1–4]. The dorsal compression of the dural sac is usually not severe, and even for severe CLSS, adipose tissue can still be seen on the dorsal side of the dural sac in axial MRI images (Fig. 6). Except for the pathogenic factors on the dorsal side of the dural sac, such as ossification of the ligamentum flavum, the leading cause of degenerative CLSS is the narrowing of the transverse diameter of the central spinal canal. Due to the cystic structure of the dural sac, the lateral pressure of the spinal canal reduces the area of the dural sac.

The primary purpose of CLSS surgical treatment is to completely decompress and minimize surgical trauma and continuous sequelae [2, 3, 9]. With the development of minimally invasive spinal surgery techniques, surgical trauma is significantly reduced, and the incidence of iatrogenic lumbar fusion surgery is reduced. MED-assisted unilateral laminectomy and bilateral decompression have obvious advantages over traditional laminectomy [11, 13], but still have the disadvantages of inevitable muscle anatomy and difficulty in decompressing the contralateral side completely [27]. Endoscopic bilateral decompression through a unilateral interlaminar approach has the advantages of small trauma, less bleeding, and less damage to ligaments and joint structures. Many scholars have used percutaneous endoscopic interlaminar decompression (PEID) to treat CLSS and achieved excellent results in the literature [15, 16].PEID has become an excellent minimally invasive surgical decompression technique for CLSS. However, this technology also has some limitations. In the first place, PEID requires general anesthesia, but elderly patients usually have multiple comorbidities and cannot tolerate general anesthesia. Secondary, PEID is difficult to completely decompress the contralateral side, especially the ventral side of the contralateral nerve root. Additional, for patients with CLSS, especially in elderly patients, lateral recess stenosis and intervertebral foramen stenosis often coexist, usually accompanied with bilateral symptoms. But PEID is not sufficient for intervertebral foramen stenosis. Finally, in some elderly patients, the interlaminar space is often narrowed by hypertrophic osteophytes and ossified ligamentum flavum. Decompression through interlaminar approach requires a large amount of time to remove the dorsal bone and the ligamentum flavum, especially in the upper lumbar segments with small interlaminar windows.

Some authors have also reported good clinical results of endoscopic lumbar unilateral decompression for lateral recess stenosis through a transforaminal approach [17, 21, 28, 29]. However, transforaminal unilateral decompression is often insufficient for CLSS. In 2019, Bao [30] reported that PETD treats central spinal stenosis, which is different from our research. Their study ruled out the pathological factor of bony central spinal stenosis and calcified disc herniation. And they only performed unilateral decompression with a unilateral approach. In contrast, our study included pathological factors of ossification compression and performed bilateral decompression. The main pathogenic factors of degenerative CLSS are the hypertrophic facet joints, hypertrophy and ossification of the ligamentum flavum, and disc herniation, usually accompanied by bilateral lateral recess stenosis. Therefore, bilateral decompression is necessary, especially for patients with bilateral buttock and lower extremity pain. The usage of deep-restricted trephine can be safer and more effective in removing the bone structure, which can significantly reduce the operation time. In our study, the operating time of bilateral decompression was still lower than their unilateral approach (116.4 min vs. 161 min).

The transforaminal bilateral approach can effectively relieve the lateral and ventral compression of the dural sac. In this study, the area of the ​​postoperative dural sac was significantly improved compared with preoperative (74.28 ± 13.08 mm^2^ vs.104.45 ± 12.51 mm^2^), and the patient’s symptoms improved significantly. These results indicated that transforaminal endoscopic surgery can also effectively decompress CLSS. In this study, we were enrolled in patients with radicular symptoms and intermittent claudication, which were caused by the compression of the dural sac and nerve roots due to degenerative changes of the lumbar spine. For CLSS, the working target area of the operation is the entire spinal canal, which requires removal of the epiphysis, intervertebral disc and ligamentum flavum which compress the dural sac and nerve roots. In order to get enough decompression space, the second foraminoplasty or even multiple foraminoplasties are always required. The specially designed deep-restricted trephine combined with a high-speed drill can make foraminoplasty more efficient and safe. For patients with CLSS, the decompression needs to reach the dorsal side of the dural sac (Fig. 3). The signs of adequate decompression are the following: the dural sac is well filled, and normal pulsation is restored, the dural sac has clear and adequate peripheral clearance.

In this study, pain in the buttocks and lower extremities improved significantly after surgery and continued to improve during the follow-up. The area of the ​​postoperative dural sac was significantly improved compared with preoperative. At the same time, the patient’s ODI and MacNab criteria were significantly improved compared with preoperative. These results are comparable to other endoscopic decompression techniques (Table 2). In this study, the average age of patients was 72 years, and 80.9% (38/47) of patients combined with various medical diseases. The average age and the incidence of preoperative medical diseases were higher than other reports, but ultimately those clinical parameters such as VAS and ODI showed significant improvements [16, 27, 31–33]. These results indicate that the advantages of endoscopic decompression through bilateral transforaminal approach not only have a good cosmetic appearance and anesthesia tolerance, but also can quickly and effectively relieve pain. The above advantages are mainly attributed to the minimally invasive endoscopic procedure. In addition, this technique of percutaneous endoscopic bilateral decompression can provide a better treatment option for elderly or medically compromised patients who are challenging to perform general anesthesia.

**Table 2:** Comparison of surgical results with endoscopic bilateral decompression for lumbar spinal stenosis

Investigator&year	No. of cases	Follow-upFU duration, months	Operative time, min	Hospital stay, d	Macnab outcome grade	Comlication rate,%	Comlication details
Komp et al. [14], 2011	74	24	44	–	subjective satisfaction 86.5%	16.21%(12/74)	transient dysesthesia 5;
							urinary retention 2;
							dural tear 2;
							revision surgery 2
							other 1;
Eum et al. [24], 2016	58	13.8	68.9	–	good-to-excellent 83% (25/30)	19.0%(11/58)	dural tear 2
							Postop headache 3
							Transient leg numbness 2
							Postop hematoma 1
							Conversion to open surgery 3
Torudom et al. [25], 2016	30	24–36	98.3	3.16	good-to-excellent 81.0% (47/58)	9.6%(3/30)	transient paresthesia 2
							Revision surgery 1
Kim et al. [26], 2017	48	7.75	–	–	good-to-excellent 96% (46/48)	12.5%(6/48)	Dural tear 3
							Revision surgery 2
							Conversion to open surgery 1
Lee et al. [27], 2018	213	26.45	105.3	2.45	good-to-excellent 93.8% (200/213) (46/48)	10.80%(23/213)	transient dysthesia12
							motor weakness 3
							dural tear 6
							Reoperations 2
Present study	47	13.6	116.4	2.6	good-to-excellent 97.9% (46/47)	10.6%(5/47)	Transient paresthesia 3
							Dural tear 2

It has been reported that incomplete decompression is one of the major drawbacks of minimally invasive bilateral decompression through a unilateral interlaminar approach, especially in the contralateral nerve root ventral decompression [27]. This is due to the limited visibility of the operating device and the limited physical space. Percutaneous endoscopic transforaminal bilateral decompression can avoid this problem perfectly. The advantages of the transforaminal approach can better deal with lateral and ventral compression of the dural sac. Although the operation time in this study is slightly longer than other decompression methods [16, 27, 31–33], the entire spinal canal including bilateral intervertebral foramina, lateral recesses and central spinal canal can be effectively decompressed through the bilateral transforaminal approach. And bilateral nerve roots and dural sac can be fully decompressed which ensure the surgical decompression effect. Some researchers have already identified that limited visualization of neural structures in endoscopic surgery may result in a higher rate of dural incision or nerve damage [27]. However, compared with previous studies of endoscopic decompression techniques, the incidence of surgical-related complications in this study was not high (10.6%). (Table 2). There were 2 dural tears (4.2%) in the current study, which is comparable to previous studies. Both dural tears were small cracks without special treatment. At present, it is still controversial whether the dural sac tear needs to be repaired in the spinal endoscopic decompression surgery [34–36]. We are more inclined to conservative views. The dural tear under endoscopy is a small crack. If there is no cauda equina hernia, the dural tears do not need repair. Because the surgical trauma is little and the muscle is not damaged, only the incision needs to be sutured tightly, and a closed cavity will be created locally. In our cases, there were no cases of cerebrospinal fluid leakage, infection, or negative consequences such as revision surgery. Three patients of transient postoperative dysthesia were present in this study, similar to other literature [16, 27, 31–33]. Postoperative dysthesia is considered to be related to the burning of the radiofrequency electrode around the nerve roots, so high-intensity radiofrequency electrode should be avoided around the nerve structures. The continuous intraoperative saline flushing provides a safer space for the differentiation of the nerve structure from the surrounding structure during endoscopic decompression. Careful operation and necessary pre-hemostasis to ensure a clear surgical area can effectively reduce complications during the operation. The use of deep-restricted trephine combined endoscopic drill can not only reduce radiation exposure, but also remove bone structure more effectively, while reducing the risk of dural tear and nerve damage.

Biomechanical studies have suggested that a 50% retention of both facet joints is necessary to preserve stability [37]. Osman SG studied the lumbar flexibility and pathological anatomy changes after posterior and transforaminal decompression [38]. The anterior medial third of the superior facet, the anterior portion of the inferior facet, and the joint part between them were resected after transforaminal decompression. Spinal flexibility changes were not observed after transforaminal decompression while the flexibility of extension and axial rotation was obviously increased after posterior decompression. The transforaminal decompression technique used in our study was similar to the above study. Even after bilateral transforaminal decompression, no iatrogenic segmental instability was recorded during the follow-up. Because the posterior structures, including spinous processes, ligaments and lamina were intact, transforaminal decompression had less damage to facet joints, which had less impact on the integrity of the spinal anatomy. The risk of iatrogenic instability caused by this surgical technique was minimized.

Compared with other MIS procedures, bilateral transforaminal decompression has the following advantages. In the first place, it can achieve adequate decompression of the central spinal canal, bilateral lateral recesses and intervertebral foramina, providing a new treatment strategy for CLSS. Moreover, decompression through the transforaminal approach can minimize the damage of facet joints, and there is no damage to the posterior ligament structure and muscles, thereby reducing iatrogenic segmental instability and avoiding spinal fusion surgery. Last but not least, this procedure can be performed under local anesthesia with high patient tolerance, and it has the advantages of MIS surgery such as less trauma, less bleeding, and faster recovery, which can provide a better treatment option for elderly or medically compromised patients who are challenging to perform general anesthesia.

## Conclusion

Except for the main pathogenic factors on the dorsal side of the dural sac, percutaneous endoscopic decompression through a bilateral transforaminal approach is sufficient for CLSS, which provides a novel minimally invasive surgical treatment for CLSS. It is a feasible, safe, and clinically effective minimally invasive procedure, especial for elderly or medically compromised patients. However, this study has many limitations. This is a retrospective study with small sample size and no control group. The follow-up time is relatively short, and long-term clinical results cannot be assessed. In the future, it is hoped that a multi-center, large-sample, and long-term follow-up randomized controlled trial will be conducted to compare bilateral transforaminal decompression and other surgical techniques for degenerative central spinal canal stenosis.

## Data Availability

The datasets analysed in this article are available from the corresponding author on reasonable request.

## Abbreviations

LSS: Lumbar spinal stenosis
CLSS: Central lumbar spinal stenosis
MIS: Minimally invasive surgery
MED: Micro-endoscopic decompression
PEID: Percutaneous endoscopic interlaminar decompression
PETD: Percutaneous endoscopic transforaminal decompression
ODI: Oswestry Disability Index
VAS: Visual Analog Scale
